# Rates, Predictors, and Barriers of Deprescribing Antipsychotics for Behavioral Symptoms in Dementia

**DOI:** 10.1093/geroni/igaf122.358

**Published:** 2025-12-31

**Authors:** Yichi Zhang, Chun-Ting Yang, James Wilkins, Dae Hyun Kim, Joshua Lin

**Affiliations:** Brigham and Women’s Hospital, Boston, Massachusetts, United States; Brigham and Women’s Hospital, Boston, Massachusetts, United States; McLean Hospital, Belmont, Massachusetts, United States; Hebrew SeniorLife, Boston, Massachusetts, United States; Brigham and Women’s Hospital, Boston, Massachusetts, United States

## Abstract

Antipsychotic medications (APMs) are commonly used to manage behavioral and psychological symptoms in dementia (BPSD), but it is recommended that APMs should be discontinued in a timely fashion when possible. We aimed to investigate the discontinuation rates, predictors, and barriers of discontinuing APMs for BPSD. We used Optum Clinformatics Data Mart database (01/01/2005 to 08/31/2024). Eligible patients were adults aged ≥65 years without prior psychiatric disorders taking APM continuously for ≥3 months, with a dementia diagnosis prior to the use of APM. The primary outcome was APM discontinuation, defined as an absence of refill for >30 days. We estimated cumulative incidence of discontinuation by calculating one minus the Kaplan-Meier estimate at 1, 3, 6, 12, and 24 months following the index date. Our study included 15,886 patients, with 524 (3.3%) haloperidol users (mean [SD] age, 82.94 [5.33] years; 327 women [62.4%]) and 15,362 (96.7%) atypical APM users (mean [SD] age, 82.18 [5.70] years; 9575 women [62.3%]). The cumulative incidence of discontinuation at 6 months of follow-up was 30.4% (95% CI, 29.5%-31.2%) among atypical APM users and 48.6% (95% CI, 43.0%-53.7%) among haloperidol users (P < .001 for the difference between haloperidol vs. atypical APMs). In the multivariate-adjusted Cox proportional hazard model, use of haloperidol, venous thromboembolism, Black or Asian race, and use of antiarrhythmics were associated with a significantly higher rate of APM discontinuation, and alcohol abuse or dependence, hip/pelvic fracture, use of anticoagulants, and use of dementia drug were associated with a significantly lower rate of APM discontinuation.

